# Supplementary material for: Cross-cultural adaptation and validation of The Resilience Scale for Kidney Transplantation (RS-KTPL) in a Chinese population
Source: PLoS One. 2025 Jul 8;20(7):e0327035. doi: 10.1371/journal.pone.0327035 (PMC12237017; doi:10.1371/journal.pone.0327035)
Supplement: S1 File — (DOCX) [file pone.0327035.s002.docx]

### ****中文版肾移植心理弹性量表（C-RS-KTPL）****

#### **量表内容：**（采用4点Likert量表：1=非常不同意，2=不同意，3=同意，4=非常同意）

| **因子分类** | **题号** | **题目内容** | **评分** |
| --- | --- | --- | --- |
| ****积极心态**** | 1 | 我积极地接受现状。 | 1 2 3 4 |
|  | 2 | 我有信心克服困难。 | 1 2 3 4 |
|  | 3 | 我把困难当作挑战。 | 1 2 3 4 |
|  | 4 | 我相信我能给别人带来希望。 | 1 2 3 4 |
|  | 5 | 我相信只要克服挑战，就能实现自己的梦想。 | 1 2 3 4 |
|  | 6 | 我能在日常生活中获得成就感。 | 1 2 3 4 |
|  | 7 | 通过解决问题，我拥有了积极的自我意识。 | 1 2 3 4 |
|  | 8 | 我关注自己的内心，并进行反省。 | 1 2 3 4 |
|  | 9 | 我有信心克服肾移植带来的挑战（副作用、并发症）。 | 1 2 3 4 |
|  | 10 | 我对未来充满信心。 | 1 2 3 4 |
| ****医疗管理**** | 11 | 我根据医生的处方调整免疫抑制剂等药物的剂量。 | 1 2 3 4 |
|  | 12 | 我严格遵守免疫抑制剂用量计划。 | 1 2 3 4 |
|  | 13 | 医护人员的鼓励和安慰给了我力量。 | 1 2 3 4 |
|  | 14 | 我会定期与医护人员沟通，以评估和管理我的症状。 | 1 2 3 4 |
|  | 15 | 我相信医护人员和他们的医疗技术。 | 1 2 3 4 |
| ****社会支持**** | 16 | 我努力履行我作为家庭成员的职责。 | 1 2 3 4 |
|  | 17 | 我觉得在家人的支持下，我已经成长为一个有价值的人。 | 1 2 3 4 |
|  | 18 | 我通过与他人的相处来获得内心的稳定。 | 1 2 3 4 |
|  | 19 | 我通过与他人的交往建立起了稳定的关系。 | 1 2 3 4 |
| ****健康习惯**** | 20 | 我始终坚持健康的饮食。 | 1 2 3 4 |
|  | 21 | 我保持稳定的体重。 | 1 2 3 4 |
|  | 22 | 我定期锻炼。 | 1 2 3 4 |

**量表说明**

**计分方式**：所有题目均采用正向计分，总分范围22-88分，分数越高表示心理弹性水平越高。

**因子说明：**

**积极心态**：评估患者面对疾病时的积极心理状态

**医疗管理**：评估患者对医疗方案的依从性

**社会支持**：评估患者获得的社会支持系统

**健康习惯**：评估患者的健康行为习惯
